# A taste for words and sounds: a case of lexical-gustatory and sound-gustatory synesthesia

**DOI:** 10.3389/fpsyg.2013.00775

**Published:** 2013-10-23

**Authors:** Olympia Colizoli, Jaap M. J. Murre, Romke Rouw

**Affiliations:** Department of Psychology, Brain and Cognition, University of AmsterdamAmsterdam, Netherlands

**Keywords:** synesthesia, fMRI, priming, perception, memory, gustation, olfaction

## Abstract

Gustatory forms of synesthesia involve the automatic and consistent experience of tastes that are triggered by non-taste related inducers. We present a case of lexical-gustatory and sound-gustatory synesthesia within one individual, SC. Most words and a subset of non-linguistic sounds induce the experience of taste, smell and physical sensations for SC. SC's lexical-gustatory associations were significantly more consistent than those of a group of controls. We tested for effects of presentation modality (visual vs. auditory), taste-related congruency, and synesthetic inducer-concurrent direction using a priming task. SC's performance did not differ significantly from a trained control group. We used functional magnetic resonance imaging to investigate the neural correlates of SC's synesthetic experiences by comparing her brain activation to the literature on brain networks related to language, music, and sound processing, in addition to synesthesia. Words that induced a strong taste were contrasted to words that induced weak-to-no tastes (“tasty” vs. “tasteless” words). Brain activation was also measured during passive listening to music and environmental sounds. Brain activation patterns showed evidence that two regions are implicated in SC's synesthetic experience of taste and smell: the left anterior insula and left superior parietal lobe. Anterior insula activation may reflect the synesthetic taste experience. The superior parietal lobe is proposed to be involved in binding sensory information across sub-types of synesthetes. We conclude that SC's synesthesia is genuine and reflected in her brain activation. The type of inducer (visual-lexical, auditory-lexical, and non-lexical auditory stimuli) could be differentiated based on patterns of brain activity.

## Introduction

*Synesthesia* refers to the experience of cross-modal sensory (and conceptual) mappings, in which the corresponding external stimulation of the additional perceived sense is absent. The term *lexical-gustatory* synesthesia has been used to refer to the automatic and consistent experience of complex taste induced by spoken and written language (Pierce, 1907; Ferrari, 1910; Ward and Simner, 2003; Ward et al., 2005; Simner and Ward, 2006; Gendle, 2007; Simner and Logie, 2008; Simner and Haywood, 2009; Jones et al., 2011; Richer et al., 2011). We present a case study on a rare form of synesthesia in participant SC. SC reported consistently and automatically experiencing tastes, smells, and feelings of texture in her mouth and throat upon hearing, speaking and reading language in addition to hearing many musical and environmental sounds. Upon investigation into SC's synesthesia, it appeared that her synesthetic experiences were similar to those previously reported (Ward and Simner, 2003; Ward et al., 2005; Simner and Haywood, 2009; Richer et al., 2011).

The prevalence of synesthesia is estimated to be about 4% of the general population (Simner et al., 2006). The prevalence of lexical-gustatory synesthesia is unknown, but may be estimated to be less than 0.2% of the population, as it was not found when a random sample was tested for the presence of different types of synesthesia (Simner et al., 2006). For a summary of reported cases of synesthesia related to taste, we refer the reader to Ward and Simner (2003). Lexical-gustatory types of synesthesia have been reported in scientific journals since at least 1907 (Pierce, 1907). In lexical-gustatory synesthesia, the synesthetic percept of taste is typically as complex as veridical taste (e.g., potatoes with gravy), while generic tastes (e.g., bitter, sweet) are notably absent (Pierce, 1907; Ward and Simner, 2003; Richer et al., 2011). We follow the suggestion of Pierce (1907) when he said that in this case *gustatory* must be taken to mean any experiences in or related to the mouth, including pressure and texture in addition to tastes and smells. SC's synesthetic experiences are commonly not only tastes, but also smells and non-olfactory related sensations of objects in the mouth and throat. As is the case with all sub-types of synesthesia, individual differences in the specific experiences are reported and multiple types of synesthesia may co-exist within one individual (e.g., Beeli et al., 2005; Hänggi et al., 2008).

It is not entirely clear how many of the previous reported cases of lexical-gustatory synesthesia co-occurred with some form of non-linguistic sound-gustatory synesthesia. Pierce, (1907) participant did not report having been aware that non-vocal sounds induced taste. However, when systematically tested, she reported experiencing synesthesia elicited by non-vocal sounds, although it was notably less specific than those elicited by words. Richer et al. (2011) reported that non-verbal sounds induced gustatory sensations in synesthete PS, such as the sound of keys on a keyboard (tasted like tomatoes). Beeli et al. (2005) presented a case of (colored) tone interval-taste synesthesia in a trained female musician, who also reported having generic tastes for certain tone intervals while she reported having specific tastes for others. Ward and Simner's, (2003) participant, JIW, who has been extensively tested on his lexical-gustatory synesthesia, reported that he did not experience synesthesia for environmental sounds.

Although inter-individual differences between lexical-gustatory synesthetes exist, there is still evidence for common mechanisms between these types of synesthetes. Phonology plays an important role linking words with synesthetic tastes, because similar sounding words tend to taste alike, more so than being visually similar (Ward and Simner, 2003). There is a growing body of evidence suggesting that word-taste pairs are linked by conceptual, semantic and learned experiences as well as by their phonology (Ward et al., 2005; Simner and Ward, 2006; Gendle, 2007; Simner and Haywood, 2009; Richer et al., 2011). The importance of the conceptual level of connection between words and tastes points toward more complex relationships than would be expected if phonology alone were the connection between words and their synesthetic tastes.

A stimulus that elicits a synesthetic experience is termed an *inducer*, and the corresponding synesthetic percept is the *concurrent* (Grossenbacher and Lovelace, 2001). External stimulation corresponding to the synesthetic concurrent is by definition absent in synesthetes. Synesthesia is often unidirectional: inducers elicit synesthetic concurrent percepts, but when the same synesthetic individual is presented with stimuli corresponding to the concurrent sense, no percepts of the inducers are experienced. Typically, words will induce taste concurrents, but tastes will not induce distinct words for lexical-gustatory synesthetes (Ward et al., 2005). A case of bidirectional lexical-gustatory synesthesia was reported by Richer et al. (2011). For this synesthete PS, some tastes evoked one or several inducer words, such as thinking of the name *Valery* while eating celery. In contrast, SC cannot name words that are associated to given tastes, with the exception of some words that have more obvious emotional significance (e.g., remembering the words that produce disgusting tastes or textures). It may be the case that bidirectional effects are typically only present under the conscious threshold in lexical-gustatory synesthetes. There is evidence showing that in grapheme-color synesthesia, bidirectional effects of colors on graphemes are present in synesthetes at a sub-conscious level (Cohen Kadosh et al., 2005, 2007; Meier and Rothen, 2007; Rothen et al., 2010). Such evidence is suggestive of mechanisms underlying structural pathways between related neural networks.

The close proximity of the primary gustatory cortex (in insular cortex) to language-related cortex (e.g., Broca's area) has prompted researchers to suggest that these areas might have additional structural connections present that give rise to the synesthetic experience (Ward and Simner, 2003). This was found to be true in the inferior temporal cortices of grapheme-color synesthetes (Rouw and Scholte, 2007) and in the insular and auditory cortices of an interval-taste and tone-color synesthete (Hänggi et al., 2008). Alternatively, indirect connections from higher-level regions may feed into these taste and language related regions of cortex due to disinhibited feedback in a different manner than in non-synesthetes, while having no differences in structural connectivity (Cytowic and Wood, 1982; Grossenbacher and Lovelace, 2001; Ward and Simner, 2003). To date there is one study known to the authors that investigated brain activation related to lexical-gustatory synesthesia (Jones et al., 2011). Synesthete JIW's brain activation was compared to a group of controls. (We note that synesthete BW was also scanned in the same study. However, BW was presented with JIW's stimuli and this makes it challenging to interpret her data). The authors highlighted the involvement of the insula in processing gustatory (Small, 2010), olfactory (Carmichael et al., 1994), and linguistic information (Dronkers, 1996; Wise et al., 1999). They found that JIW's left anterior insula showed more activation in the presence of unpleasant words compared to neutral words and this differed for controls, while the precuneal cortex showed a difference between JIW and controls when comparing “tasty” and “tasteless” words.

In the present study, we investigated brain activation related to the synesthetic experience of taste and smell for SC. We refer to the literature to determine if SC shows normal language and sound-related brain activation as a within-subject control measure, because we were unable to scan a control group to compare to SC. In addition to the functional magnetic resonance imaging (fMRI) data of SC, a behavioral priming task was designed in order to test certain assumptions based on the description of her synesthesia. For the behavioral experiment, SC's behavior was compared to a group of matched controls. We present her case information before the specific hypotheses concerning this priming experiment.

## Case history of SC

At the time of testing, SC was a 29 year-old right-handed woman. She works as a musician, performing artist and teacher. SC's native language is Dutch. SC is fluent in English (exposed since at least age 10) and is also proficient in German and French (studied in school beginning at age 13), but does not consider herself fluent in either of these languages. Languages that she does not understand do not induce synesthetic experiences. SC reported experiencing tastes, smells, textures, as well as experiences which are “hard to describe” upon hearing, reading, and thinking about words, letters and a subset of non-linguistic sounds, including music and environmental noises. Her synesthesia appears to be unidirectional, meaning that tastes and smells do not induce the experience of certain words (i.e., in general she cannot answer a question such as “which words taste like potatoes?”). She reported having experienced this type of synesthesia all her life. SC realized that she was different from others when she was 7 years old. SC reported that her mother experiences days of the week in color but was unaware of the presence of any other types of synesthesia in her family. There did not seem to be any other type of synesthesia present in SC (e.g., related to color). She did report experiencing what has become known in the non-scientific literature and on YouTube as autonomous sensory meridian response (ASMR), which is a pleasurable, specific and intense tingling feeling in the head and body upon hearing “soft” or “crackling” sounds. SC has no history of neurological disease or trauma, reported no serious health complaints or sensory deficits. Although we did not test her IQ or memory, SC reported having been an above-average student and never having any learning disabilities.

SC reported that auditory linguistic stimuli are the strongest inducers of her synesthesia, including hearing her own voice. Each letter of the alphabet and most words elicit a synesthetic percept in SC. When reading, the synesthesia is only induced when she repeats the words mentally to herself as “inner speech.” SC reported that she believes that the tastes of words are related by the phonology with the exception of food words. (Although we did not test this directly, we briefly examined this claim in relation to her multilingualism and it appeared to hold true). All food words taste like the foods they describe without any known exceptions. The types of non-linguistic sounds that induce synesthetic experiences in SC are discrete sounds, or “sounds apart from each other.” For example, a song with complex components does not necessarily induce a taste or smell, however, the sound of just a bass drum does. SC is a trained musician and did not report that certain tones, chords, or types of instruments produced consistent types of tastes or patterns of tastes.

She described the synesthetic taste as being less intense or real as veridical taste even though she does feel the synesthetic taste on her tongue and in the mouth. The tastes are located on the tongue, mainly in the back. However, the specific location can change depending on the nature of the taste. The synesthetic concurrents are overall lacking the experience of temperature. For example, the Dutch word *alsof* tastes like soft-serve ice cream, however, it does not feel cold. SC has many synesthetic olfactory percepts in addition to tastes. She described the experience of synesthetic smells as the following: “It is as if it's still in my mouth after inhaling something that has smell.” Some words and sounds induce sensations that are neither tastes nor smells, for example, the feeling of swallowing buttons, or stuffing her mouth full of marbles. These sensations are not strong enough to trigger her gag-reflex, but it does make her “a little bit nervous.” SC described each synesthetic concurrent (tastes, smells, and physical sensations) as a very fast percept. When engaged in conversation, each percept is experienced one after the other at the rate of normal speech. SC reported that the tastes of words do not mix, and compound words (e.g., *bookend*) have two distinct tastes corresponding to the component words (e.g., *book* and *end*). However, she did report that two or more tastes at the same time do occur. Although they do not create a new taste, they can co-exist, often located on distinct parts of the tongue or mouth. SC reported feeling that the presence of real food or beverages in the mouth does not affect the experience of the synesthetic concurrent sense.

## Goals of the current research

**Figure 1:**
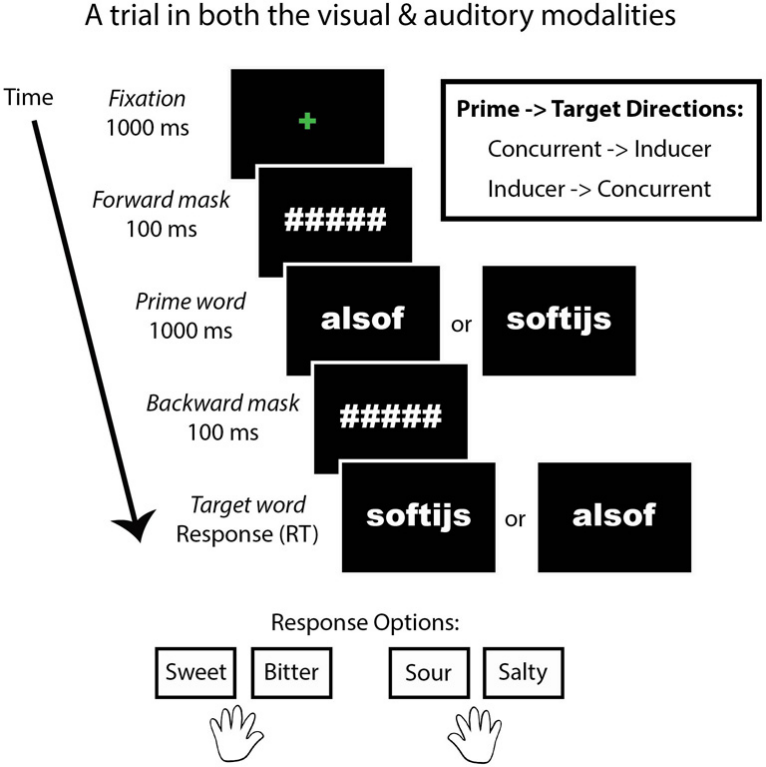
**A trial from the priming task.** This task was presented in both a visual and auditory modality. Congruency refers to whether the type of tastes of the prime and target words were congruent (e.g., sweet–sweet) or incongruent (e.g., sweet–sour). Direction refers to whether the prime and target words were presented in SC's inducer-concurrent (I-C) direction or concurrent-inducer (C-I) direction.

We designed a behavioral priming task (Figure 1) in which the presentation modality, congruency of the prime related to the target taste, and the inducer-concurrent direction were manipulated. SC reported that auditory stimulation is stronger than visual stimulation in eliciting her synesthesia and we predicted a difference in her performance between presentation modalities. In addition, we were interested to know whether Stroop-like effects of congruency (Stroop, 1935) related to the type of tastes (sweet, bitter, sour, and salty) would be evident in SC. This was possible because the concurrent words themselves taste like the foods they describe (e.g., *apple* tastes like “apple”). Although SC does not report generic tastes or smells as concurrent sensations, she was given a forced-choice task in which she had to classify each concurrent as either sweet, salty, bitter, or sour. In order to test for an effect of congruency, the relationship of the prime to the target word was manipulated in two ways: the type of taste of prime and target words was either congruent (e.g., sweet followed by sweet) or incongruent (e.g., sweet followed by sour). Lastly, we tested for bidirectional effects between the inducer and concurrent words by including two prime-target directions: SC's original inducer-concurrent direction (I-C direction) or the current-inducer direction (C-I direction). This task consisted of a 2 × 2 × 2 factorial within-subjects design. We tested for a significant difference in SC's performance compared to a group of trained controls.

Using fMRI, we tested whether we could successfully localize written and spoken language as well as non-linguistic sound-related activation in SC's brain based on the fMRI localization literature. Furthermore, we contrasted activation related to the experience of synesthesia (i.e., “tasty”) to unrelated activation (i.e., “tasteless”) in order to uncover synesthesia-related brain processes. This contrast was compared and contrasted to the case of synesthete JIW reported in Jones et al. (2011).

## Materials and methods

### Participants

SC is a right-handed woman and was 28–29 years old across the course of the testing period. SC has been educated at the university level. The control group that took part in the behavioral priming task was composed of 16 non-synesthetic participants (*M* = 29.25 years, *SD* = 1.72). Controls were matched to SC for native language, age, gender, and education. Eleven of these control participants completed the consistency test for word-taste associations (12 participants indicated that they would be available for a retest at a later unknown date. One of these participants did not respond to our further communications). None of the control participants reported experiencing lexical-gustatory synesthesia, grapheme-color synesthesia, or any other types of synesthesia, although not all types of synesthesia were exhaustively ruled out. All participants were informed that they could terminate their participation at any time and gave written informed consent before participating in the research. All participants received €20 for participating in the behavioral session and it took about 1.5 h to complete. SC received an additional €40 for filling in questionnaires about her synesthesia, which were completed outside of the lab. The fMRI experiment was done for a Dutch TV series, and SC was not compensated financially for the fMRI experiment. SC was screened with a standard magnetic resonance imaging (MRI) protocol and gave written informed consent before participating in the MRI study.

This research was approved by the Ethics Committee of the Department of Psychology at the University of Amsterdam.

### Consistency test materials and procedure

The list used for the consistency test of word-taste associations for SC consisted of 110 Dutch words taken from the CELEX database (Max Plank Institute: http://celex.mpi.nl). Both frequent and infrequent words were chosen for this list. The frequency measure used was occurrences per 1 million words. The consistency test for the controls consisted of a subset of 30 random words from SC's list.

For both SC and the controls, the 1st version of each word list was in randomized order (non-alphabetical) and the 2nd version of each list was again randomized. SC was not informed that she would be given the list again (after 9 months), while the controls were informed that they would be given a “pop-quiz” at an unknown time in the following few weeks to test their memory for these associations. The instructions for SC were to respond freely while describing the synesthetic experience and to be as detailed as possible. SC rated the intensity of the experience on a scale of 1 to 5, where 1 indicated “not intense” and 5 indicated the “most intense” experience. In addition, SC was asked to categorize the type of each taste or smell as: sweet, bitter, sour, or salty, and these ratings were used to design the stimuli for the behavioral priming task. The control group was instructed to generate taste and/or smell associations for the list of 30 words and to be as specific as possible.

### *post-hoc* classification as taste or smell

After the consistency test had been completed, SC classified each word (the 104 inducing words from the consistency test) as inducing a smell, taste, or other type of feeling. In addition, we asked her to classify each concurrent sensation as pleasant or unpleasant. She was permitted to give combinations of these categories (e.g., smell and taste).

### Priming task materials and procedure

The Dutch words and the English translations of the eight word pairs used in the behavioral task are given in Table 1. These 16 words were used as the stimuli in the behavioral task. These 8 word pairs were all identically consistent except for *durned*, which was added later in order to balance the stimuli.

**Table 1:** **Stimuli used in the behavioral priming task**.

**Word**	**Inducer**	**Concurrent**	**Taste**	**Intensity**
**pair**				
1	Mijn (mine)	Winegums (candy)	sweet	5
2	Alsof (as if)	Softijs (soft serve ice cream)	sweet	5
3	Duik (dive)	Chloorwater (chlorine water)	bitter	3.5
4	Door (by)	Rioollucht (sewage gas)	bitter	4
5	Durend (lasting)	Zuurring^*^ (sorrel)	sour	5
6	Over (about)	Maagzuur (stomach acid)	sour	4
7	Vrouw (woman)	Wokkels (potato chips)	salty	5
8	Naar (to)	Karbonades (pork chops)	salty	5

The word-taste/smell pairs used were based on SC's synesthetic associations. The closest English translations are in parentheses.

* Zuurring should be spelled as zuring, however, we kept it the way SC spelled it.

All control participants began the experiment with a computerized training task. They were instructed to learn eight pairs of associations between words and specific tastes and smells. In addition, the controls were trained to learn which type of taste or smell (sweet, bitter, sour, and salty) each of these eight pairs had. The instructions were to indicate if the pair of words on screen were “correct” or “incorrect,” meaning that they are correctly associated with each other or not. After each “correct” pair, a screen with four taste choices appeared, and participants were instructed to indicate if the pair of words was sweet, bitter, sour, or salty. They received feedback on accuracy after each response was made. The word pairs were presented in both inducer-concurrent directions (I-C and C-I). The control participants performed rounds of the training task until they reached 95% accuracy or higher by the end of a round. Thereafter, they were given a paper test in which they had to fill in the eight pairs of words correctly in a blank table as well as the corresponding taste (in the I-C direction). This was done in order to see if the participants could recall the correct associations freely. If not, they repeated a round of the training task and the free-recall test.

Each trial of the word-pair training task for the control group began with a green centered fixation cross for 1000 ms, followed by one of the word pairs, underneath which two response options were visible until a response was made: correct or incorrect (word-pair association). Feedback on accuracy was given for 1000 ms following each trial. When the pair of words was the correct association, a screen appeared thereafter asking what type of taste the word-pair association had and remained present until a response was made. The four possibilities were: sweet, bitter, sour, and salty. Feedback was given for 1000 ms on accuracy of the type of taste of the word pair. One round of the training task consisted of 160 correct-incorrect association trials and 80 type-of-taste trials. From the 160 correct-incorrect trials, 80 trials were in SC's I-C direction and 80 were in the C-I direction. The words were presented in white Arial font against a black background.

After this word-taste training, the priming experiment began. For SC, the beginning of the experiment was the priming task. The priming task consisted of two modality conditions: visual and auditory. The task was identical in the two cases, except for the modality of the word presentation. In the visual condition, two words were presented on the computer screen in rapid succession. In the auditory condition, two words were presented via headphones in rapid succession. The instructions were to indicate the taste (sweet, bitter, sour, or salty) of the second word. The first word in each trial was the prime, while the second was the target. The tastes of the first and second words were either congruent or incongruent with each other (i.e., salty followed by salty is congruent, while sweet followed by salty is incongruent). An equal number of congruent and incongruent trials were given within each modality (visual and auditory). The directionality of SC's inducing word and taste-related concurrent word could differ (i.e., the inducer followed by the concurrent vs. the concurrent followed by the inducer), and the directions (I-C vs. C-I directions) of the word-taste pairs were balanced within each modality (visual and auditory). Each participant received the same experiment, meaning that the conditions were not counterbalanced between participants in order to better compare SC to the control group. Participants started with the visual modality followed by the auditory modality. Trials belonging to word-taste congruency and inducer-concurrent directionality were pseudo-randomized within participants but not between them. The taste and response options were: sweet, bitter, sour, or salty. These four tastes corresponded to the first two and last two buttons on a seven-button response box, respectively, (the middle three buttons were unused). Participants used the index and middle fingers of each hand to respond.

In the visual condition, the beginning of a trial was indicated by a green centered fixation cross for 1000 ms, followed by a forward mask (hash tags corresponding to each letter in the prime word) for 100 ms, followed by the prime for 1000 ms, followed by a backward mask (identical to the forward mask) for 100 ms, followed lastly by the target word. The target word remained on screen until a response was made. The words were presented in Arial Black font printed in white typeface color against a black background.

In the auditory condition, the beginning of a trial was indicated by a green centered fixation cross for 1000 ms, followed by a forward mask (hash tags corresponding to each letter in the word) for 100 ms, followed by the prime for 1000 ms (via the headphones), followed by a backward mask (identical to the forward mask) for 100 ms, followed lastly by the target word (via the headphones). The target word was heard once. At the onset of the target word, a blue fixation cross appeared and remained on screen until a response was made.

There were 192 trials in each modality (visual and auditory). From these 192 trials, 96 trials were congruent and 96 trials were incongruent (e.g., a sweet prime word followed by a sweet target word was congruent, while a sour prime word followed by a sweet target word was incongruent). In addition, congruency was balanced with word-pair direction (i.e., inducer or concurrent as prime): Ninety-six trials were in the I-C word-pair direction, and 96 trials were in the C-I word-pair direction. The priming task consisted of 384 trials in total.

Before the priming task began, and after the word-pair training task was completed, participants completed a short button training task in order to be able to respond to the four different tastes as fast as possible. The button task consisted of 80 trials; 20 trials per taste. A trial began with a centered fixation cross for 500 ms, followed by one of the four taste words until a response was made or 3000 ms (missed trial). After each trial, a feedback screen appeared for 1000 ms indicating correct and incorrect responses. All stimuli were presented in white Arial font against a black background.

During the entire behavioral session, participants were seated ~42 cm in front of the computer monitor. All stimuli were presented on a PC using Presentation (version 14.1; www.neurobs.com) on a 23-inch monitor. The screen resolution was 1280 × 1024 pixels, 32-bit color depth. The refresh rate of the screen was 60 Hz. All responses were recorded with a USB 7-button box.

### fMRI procedure

Written words and auditory stimuli were presented in separate runs. SC was presented with two visual and two auditory runs, which were interleaved. SC was not instructed to make any responses in the scanner. During the visual runs, SC was instructed to actively view all the words that appeared on screen in addition to the periods when only a fixation cross was presented. Visual stimuli were projected onto a screen at the rear of the scanner. SC viewed the stimuli through a mirror placed above her head on the head coil. During the auditory runs, SC was instructed to keep her eyes closed, while paying close attention to the sounds through the headphones. SC wore earplugs and headphones, adhering to the standard MRI safety protocol, and foam pads were used to minimize head motion. The volume of the auditory stimuli was adjusted prior to scanning to ensure that she could hear all sounds over the noise of the scanner and through the ear protection. We instructed SC to ignore the scanner noises as much as possible. Before scanning, SC listened to audio recordings of typical scanner noises. SC reported that most of the scanner noises did not induce synesthesia. The few sounds that elicited synesthesia in SC were not intense, and SC reported that it was easy for her to ignore them. We verified that this was the case with SC after scanning. Importantly, scanner noises in the functional runs were the same in all conditions.

### Written language localizer

In order to localize the (VWFA) and related experiences of synesthetic taste, a 16-second blocked design with four conditions was used: (1) 80 Dutch words with four letters and equal average frequencies chosen at random; (2) The 80 words from (1) presented as Chinese characters in Hanzi Kaishu font; (3) 80 Dutch words that evoked an intense synesthetic experience for SC (all words had a rating of 4 or 5 on a 5-pt. Likert scale); (4) 10 Dutch words that evoked a weak-to-no synesthetic experience or no synesthesia at all for SC (all words had a rating of 1 on a 5-pt. Likert scale). Each of these four conditions was presented five times, for a total of 20 stimulus blocks within one run. The order of these four conditions was pseudo-randomized. Stimuli were visually presented in 16-second blocks. A 16-second baseline (rest) period was presented between each stimulus block in which a centered fixation cross was presented on screen. Sixteen trials of words were presented within each stimulus block in random order. Each trial consisted of a centered fixation cross for 500 ms, followed by a word for 500 ms. There were 320 word trials in each run (80 trials per condition). Each run lasted 11 min. Two runs of this localizer were presented to SC in the MRI scanner. A total of 640 trials were used in the final analysis (160 trials per condition). All words were presented in black Courier New font against a white background.

### Verbal and non-verbal sound localizer

In order to localize verbal and non-verbal sounds and the related experiences of synesthetic taste, a 16-second blocked design with four conditions was used: (1) Spoken Dutch audio from TV news broadcasts; (2) Spoken Turkish and Russian audio from TV news broadcasts; (3) Musical instruments (bagpipes, drums, guitar, oboe, trumpet, triangle, accordion, harmonica, and xylophone); (4) Environmental sounds (applause, rain, siren, wind, electric fan, keyboard, typewriter, and race car). Each of these four conditions was presented five times, for a total of 20 stimulus blocks within one run. The order of these four conditions was pseudo-randomized. Stimuli were presented through headphones in 16-second blocks. The auditory stimuli were continuously presented within each block, with no breaks in between the different sounds. In each environmental sounds block, each of the eight sounds was played for 2 s. In each musical instruments block, two of the ten instruments were played for 8 s. The order of the different musical instruments and environmental sounds was randomized within a block. A 16-second baseline (rest) period was presented between each stimulus block in which no sounds were heard. During the auditory runs, the screen was black with a centered white fixation cross. SC was instructed to keep her eyes closed during the entire run. Each run lasted 11 min. Two runs of this localizer were presented to SC in the MRI scanner. In this condition, one trial was considered one 16-second block. Therefore, 40 trials were included in total.

### fMRI data acquisition

Scans were acquired on a Philips 3 Tesla Achieva TX scanner, located at the Spinoza Center, Amsterdam, the Netherlands. Whole brain gradient-echo echo-planar images (voxel size = 3 × 3 × 3 mm, FOV = 240 × 240, matrix = 80 × 80, *TR* = 2000 ms, *TE* = 27.63 ms, flip angle = 76.1°, slice thickness = 3 mm, slice gap = 0.3 mm, 38 slices per volume, sensitivity encoding factor of 2) were acquired to measure blood oxygen level-dependent (BOLD) magnetic resonance images with a 32-channel SENSE head coil. Each functional run consisted of 337 volumes and lasted ~11 min.

A T1 anatomical scan was acquired (voxel size = 1 × 1 × 1 mm, FOV = 256 × 256, matrix = 256 × 256, *TR* = 8090 ms, *TE* = 3.71 ms, flip angle = 8°, slice thickness = 1 mm, no slice gap, 160 slices per volume, 1 volume was acquired that lasted ~5 min) so that functional images could be registered to native anatomical space and normalized to the Montreal Neurological Institute (MNI) standard space.

### fMRI pre-processing and statistical analysis

Analyses of the MRI images were carried out using the Oxford Centre for Functional MRI of the Brain (FMRIB) Software Library (FSL) version 5.0.4, Oxford, UK: http://www.fmrib.ox.ac.uk/fsl (Smith et al., 2004; Woolrich et al., 2009; Jenkinson et al., 2012). Statistical analyses were conducted using FSL's fMRI Expert Analysis Tool, (FEAT version 6.00). Preprocessing steps included motion correcting using MCFLIRT (Jenkinson et al., 2002), slice-timing correction (temporal sinc interpolation), pre-whitening (FILM algorithm), spatial smoothing (a 5 mm Gaussian kernel of full-width at half-maximum), grand-mean intensity normalization of the entire 4D dataset by a single multiplicative factor, and high-pass temporal filtering (cutoff at σ = 50 s). Voxels belonging to brain tissue were extracted from non-brain tissue voxels using the Brain Extraction Tool (BET; Smith, 2002). No runs were discarded due to motion or other artifacts.

In the first-level analysis, the time course of each run was composed of a blocked design convolved with the double gamma hemodynamic response function and tested with an uncorrected voxel threshold of *P* = 0.05. Resulting contrast images were linearly registered to the T1-weighted image using FLIRT with 7° of freedom and the full search space (Jenkinson and Smith, 2001; Jenkinson et al., 2002; Greve and Fischl, 2009), then spatially normalized to the T1-weighted MNI-152 stereotaxic space template (2 mm) using FNIRT with 12° of freedom and the full search space (http://fsl.fmrib.ox.ac.uk/fsl/fslwiki/FNIRT).

In the higher-level analysis, the mean of the first-level runs was computed using a fixed-effects model of variance. *Z*-statistic (Gaussianized T/F) images were thresholded using clusters determined by *Z* > 2.3 and a (corrected) cluster significance threshold of *P* = 0.05 (Worsley, 2001), controlling the family-wise error rate.

## Results

In order to compare the behavioral data of SC to a small sample of controls, we followed the methods developed and published in Crawford and Howell (1998); Crawford and Garthwaite (2002), and Crawford et al. (2010). This methodology is in essence a modified independent samples *t*-test, where the individual case is treated as a sample of *N* = 1 (thereby not contributing to the estimate of the within-group variance) and the null hypothesis is that the case's score is an observation from the control sample's distribution. This methodology was more appropriate to the present circumstance than other standard methods (e.g., comparison of *Z*-scores), because it does not treat the statistics of the normative sample as population parameters, but rather as sample statistics. With small sample sizes (*N* < 50), *Z*-scores overestimate the abnormality of the individual's case by assuming that the variance is known, when it is not, increasing Type 1 error rates (e.g., Crawford and Howell, 1998). Furthermore, by using this modified *t*-test, we avoid violating important assumptions of other widely used statistical tests, such as the assumption of data independence in the Chi-square test.

The modified *t*-test is available as open-source software from the website: http://homepages.abdn.ac.uk/j.crawford/pages/dept/SingleCaseMethodsComputerPrograms.HTM. The test used in the current study was Singlims_ES.exe. The output of this test includes hypothesis testing in the form of *t*-test statistics, a point estimate of the abnormality of the case's score as a percentage of the population falling below it, confidence intervals associated with the uncertainty of the abnormality estimate, effect size (*Z*_cc_), and confidence intervals associated with the uncertainty of the effect size (effect size is comparable to Cohen's *d*; see Crawford et al., 2010).

The significance level was set at α = 0.05. As our comparisons between SC and the control group involved multiple *t*-tests, we corrected for multiple comparisons using the Bonferroni method.

### Consistency of sc's word-taste associations

The 1st and 2nd versions of the word lists were completed by SC with a period of 9 months in between tests. Although all words were in the Dutch language, SC gave her answers in English on both versions of the list. Following Gendle (2007), we computed consistency based on different levels of identity: identical, nearly identical, conceptually related, and unrelated. We feel that this method is more sensitive to the complexity of the synesthetic experiences and allows for a better understanding of the consistency score.

Of the 110 words presented to SC, 94.5% of the words induced a synesthetic experience. Six words (5.5%) did not induce a synesthetic experience in either the first or second list. We did not include these six words in the calculation of the percentage of consistent answers given. Therefore, the consistency scores given below were calculated based on 104 words. If SC gave an indication of taste/smell in one of the lists, but not in the other, this was counted in the consistency analysis as an unrelated response.

**Figure 2:**
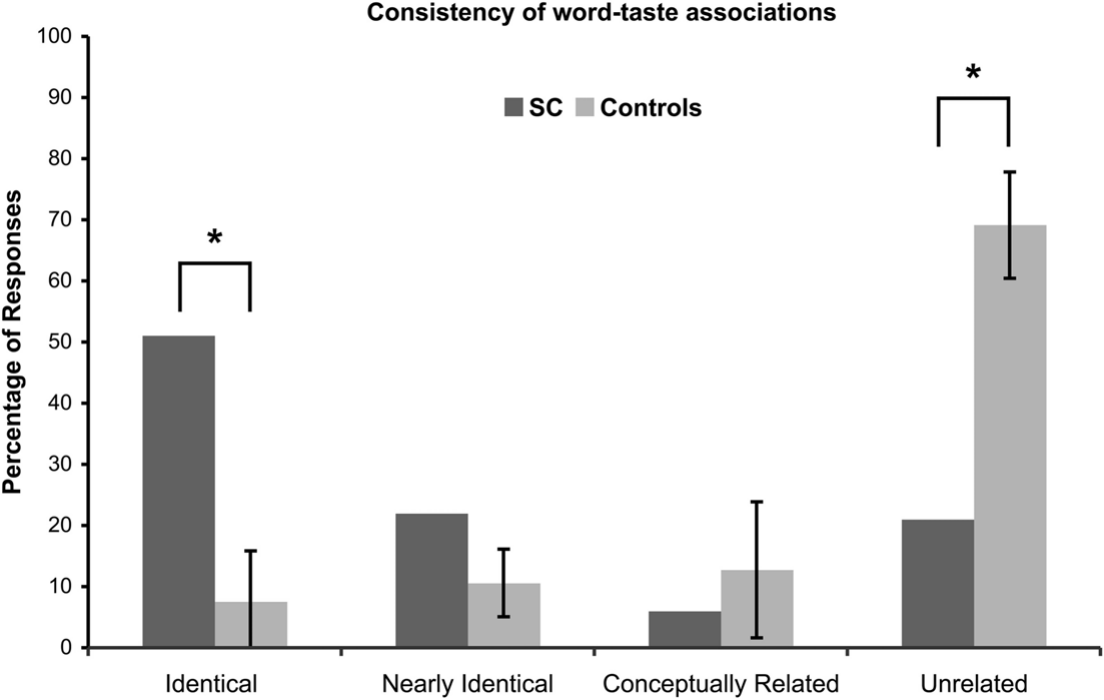
**Consistency of word-taste associations.** A significant difference is denoted with by (*) and corrected for multiple comparisons.

Four different scores for consistency were calculated based on the four levels of response categorization (Figure 2): Identical responses, 53/104 = 51%; Nearly identical responses, 23/104 = 22%; Conceptually related responses, 6/104 = 6%; Unrelated responses, 22/104 = 21%.

A majority of the words (51%) had identical responses, 73% of the words had identical and nearly identical responses (cumulative), and 79% of the words had identical, nearly identical, and conceptually related responses (cumulative), while 21% of the words had unrelated responses.

The 22 instances of unrelated responses consisted of three words (2.7%) that induced “hardly anything” in the first list only, seven different words (6.4%) that induced “hardly anything” in the second list only, and twelve words (11%) that induced two unrelated synesthetic experiences between the first and second lists.

The intensity ratings of the identical and nearly identical responses between the two lists were positively correlated, *r*_s(74)_ = 0.49, *p* < 0.001, indicating that if a word induced an intense experience while filling in the list the first time, it also induced an intense experience while filling in the second list, and vice versa. This result was the same when correlating the intensity ratings of all words, including the six words that never induced a synesthetic experience an intensity rating of 1 in both lists, *r*_s(108)_ = 0.60, *p* < 0.001. The intensity ratings of these 22 unrelated words were also positively correlated, *r*_s(20)_ = 0.46, *p* = 0.032, indicating that if a word induced an intense experience while filling in the first list, it also induced an intense experience while filling in the second list even if the description of the synesthetic experiences were unrelated.

### Consistency of the control group's word-taste associations

The 1st and 2nd version of the lists were completed with a period of three to four weeks in between tests. Four different scores for consistency were calculated based on the four levels of response categorization (*N* = 11; Figure 2): Identical responses, 2.27/30, *M* = 7.57% (*SD* = 8.31); Nearly identical responses, 3.18/30, *M* = 10.60% (*SD* = 5.54); Conceptually related responses, 3.82/30, *M* = 12.73% (*SD* = 11.14); Unrelated responses, 20.73/30, *M* = 69.10% (*SD* = 8.70).

A minority of the words (7.57%) had identical responses, 18.17% of the words had identical or nearly identical responses, and 30.90% of the words had identical, nearly identical, or conceptually related responses, while 69.09% of the words had unrelated responses.

### Is SC more consistent than the control group?

A comparison between SC and the controls' consistency scores are given in Figure 2. If SC's synesthesia is genuine, we expected SC to have significantly more identical responses and significantly less unrelated responses compared to the controls (in percentages). Results are presented in Table 2. As expected, SC's score for identical responses was significantly greater than that of the control group. In addition, SC's score for unrelated responses was significantly lower than that of the control group. No significant differences in scores were found between SC and the controls for nearly identical or conceptually related responses.

**Table 2:** **Differences in consistency scores for word-taste associations between SC and controls**.

**Type of response**	**Control sample**		**SC's score (%)**	**Significance**		**Estimated % of the**		**Estimated effect**	
	**(***N* = 11**)**			**test**		**control population**		**size (*Z_cc_***)	
						**obtaining a lower score**			
						**than the case**			
	**Mean (%)**	***SD***		***t***	***p***	**Point**	**(95% *CI*)**	**Point**	**(95% *CI*)**
Identical	7.58	8.31	51	5	0.000^*^	99.97	(88.80 to 100.00)	5.23	(2.89 to 7.55)
Nearly identical	10.61	5.54	22	1.97	0.039	96.13	(83.52 to 99.91)	2.06	(0.98 to 3.12)
Conceptually related	12.73	11.14	6	−0.58	0.288	28.79	(10.79 to 52.17)	−0.6	(−1.24 to 0.05)
Unrelated	69.09	8.70	21	−5.29	0.000^*^	0.02	(0.00 to 0.12)	−5.53	(−7.98 to −3.07)

*A significant difference (1-tailed) is indicated* (

* ) and corrected for multiple comparisons.

SC completed a word-taste association list that was more than three times as long as that of the controls. Furthermore, SC's retest was given 9 months after the first test, while the control group was given their retest within three to four weeks after the first test. SC was not informed that we would retest her word-taste associations, while the control group was instructed to remember the associations they gave. SC gave more identical responses than the control group, while the control group gave more unrelated responses compared to SC. The nearly identical and conceptually related responses did not differ between SC and the control group. Given the above, we conclude that SC's word-taste associations are significantly more consistent than that of the control group, implying that her synesthesia is highly consistent over time and therefore, genuine.

### *post-hoc* classification as taste or smell

SC classified 104 words as inducing a smell, taste or other type of experience, in addition to it being a pleasant or unpleasant experience.

SC classified 60/104 (57.69%) words as taste: 46 (76.67%) were rated as pleasant and 14 (23.33%) as unpleasant. The majority of tastes induced were pleasant to SC.

SC classified 33/104 (31.73%) words as smell: 12 (36.36%) were rated as pleasant and 21 (63.64%) as unpleasant. The majority of smells induced were unpleasant to SC.

SC classified 6/104 (5.77%) words as “other” (indicating a physical feeling or bodily sensation): 3 (50%) were rated as pleasant and 3 (50%) as unpleasant. These sensations were equally likely to be pleasant or unpleasant to SC.

SC classified 1/104 (0.96%) words as inducing both smell and taste. This word was rated as both pleasant and unpleasant.

SC classified 4/104 (3.85%) words as inducing both a taste and “other.” These four words (100%) were all rated as unpleasant.

In total, 61/104 (58.65%) words were rated as inducing a pleasant experience, 42/104 (40.39%) as unpleasant, and 1/104 (0.96%) as both pleasant and unpleasant simultaneously (SC explained that this word can induce two distinct sensations depending on the context). The majority of the words tested induced a pleasant experience to SC.

### Priming task

The following abbreviations are used: *M* = mean, *SD* = standard deviation from the mean (*N* = 16), RT = reaction times. Only correct trials were included in the RT analyses. All RT data is reported in milliseconds. The significance level used was α = 0.05.

### Trained control group

A 2 × 2 × 2 repeated measures ANOVA was carried out on the data of the control group (i.e., SC's data was not included in the following repeated measures analysis). The factors of interest were: modality (visual vs. auditory), congruency (incongruent vs. congruent), and inducer-concurrent direction (I-C vs. C-I).

**Figure 3:**
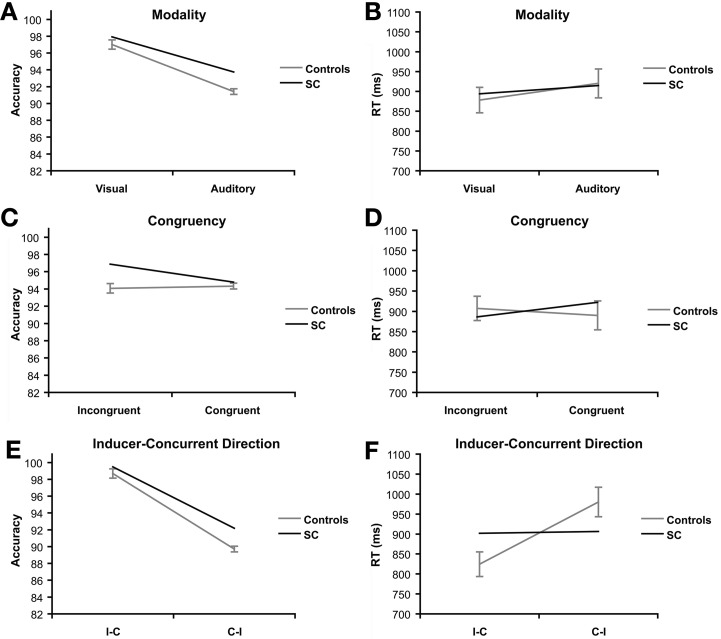
**Priming task: main effects of synesthete SC and controls (*N* = 16).** Main effects are plotted for three factors of interest: modality refers to the visual or auditory presentation of the stimuli. Effects of modality are presented for accuracy **(A)** and reaction time **(B)**. Congruency refers to whether the type of tastes of the prime and target words were congruent (e.g., sweet–sweet) or incongruent (e.g., sweet–sour). Effects of congruency are presented for accuracy **(C)** and reaction time **(D)**. Direction refers to whether the prime and target words were presented in SC's inducer-concurrent (I-C) direction or concurrent-inducer (C-I) direction. Effects of inducer-concurrent direction are presented for accuracy **(E)** and reaction time **(F)**.

There was a significant effect of modality on accuracy scores, *F*_(1, 15)_ = 165.97, *p* < 0.001, η_p_^2^ = 0.917 (Figure 3A). Controls scored higher on visual trials (*M* = 97.01%, *SE* = 2.19) compared to auditory trials (*M* = 91.41%, *SD* = 1.35). A trend was found in RT for the effect of modality, *F*_(1, 15)_ = 3.20, *p* = 0.094, η_p_^2^ = 0.18 (Figure 3B). Controls were faster on visual trials (*M* = 878.05 ms, *SD* = 127.52) than auditory trials (*M* = 920.28 ms, *SD* = 145.61), but this difference failed to reach significance in RT.

There was no evidence of a congruency effect in RT or accuracy in the controls (*F* < 1.5; Figures 3C,D).

A significant effect of direction was found in both accuracy, *F*_(1, 15)_ = 344.70, *p* < 0.001, η_p_^2^ = 0.96 (Figure 3E), and RT, *F*_(1, 15)_ = 51.43, *p* < 0.001, η_p_^2^ = 0.77 (Figure 3F). The controls were more accurate (*M* = 98.70%, *SD* = 1.40) and faster (*M* = 824.24 ms, *SD* = 123.08) in the I-C direction compared to the C-I direction (*M* = 89.71%, *SD* = 2.24; *M* = 980.08 ms, *SD* = 147.72).

**Figure 4:**
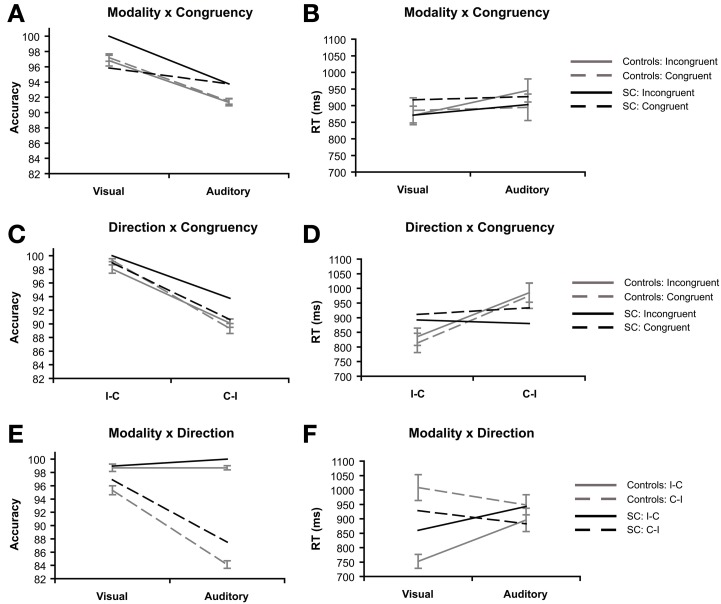
**Priming task: 2 × 2 interaction effects of synesthete SC and controls (*N* = 16).** Interactions are plotted for three factors of interest: modality refers to the visual or auditory presentation of the stimuli. Congruency refers to whether the type of tastes of the prime and target words were congruent (e.g., sweet–sweet) or incongruent (e.g., sweet–sour). Direction refers to whether the prime and target words were presented in SC's inducer-concurrent (I-C) direction or concurrent-inducer (C-I) direction. The interaction between modality and congruency are presented for accuracy **(A)** and reaction time **(B)**. The interaction between inducer-concurrent direction and congruency are presented for accuracy **(C)** and reaction time **(D)**. The interaction between modality and inducer-concurrent direction are presented for accuracy **(E)** and reaction time **(F)**.

The interaction between modality and congruency was significant for RT, *F*_(1, 15)_ = 11.72, *p* = 0.004, η_p_^2^ = 0.44 (Figure 4B), but not in accuracy (*F* < 1; Figure 4A). The congruency effect in RT (incongruent vs. congruent) was bigger in the auditory condition (*M* = 50.85 ms, *SD* = 65.31) than the visual condition (*M* = −14.86 ms, *SD* = 75.27).

The interaction between direction and congruency was marginally significant in accuracy, *F*_(1, 15)_ = 4.37, *p* = 0.054, η_p_^2^ = 0.23 (Figure 4C), while no interaction was found in RT (Figure 4D). The difference between directions (I-C vs. C-I) was bigger on congruent (*M* = 10.03%, *SD* = 3.04) trials compared to incongruent trials (*M* = 7.94%, *SD* = 2.49).

The interaction between modality and direction was significant in both accuracy *F*_(1, 15)_ = 259.20, *p* < 0.001, η_p_^2^ = 0.945 (Figure 4E), and RT, *F*_(1, 15)_ = 35.23, *p* < 0.001, η_p_^2^ = 0.70 (Figure 4F). In accuracy, the difference between directions (I-C vs. C-I) was bigger in the auditory condition (*M* = 14.58%, *SD* = 2.47) compared with the visual condition (*M* = 3.39%, *SD* = 2.30). This pattern is reversed in the RT data, the difference between directions (C-I vs. I-C) was bigger for visual trials (*M* = 255.65 ms, *SD* = 130.36) compared to auditory trials (*M* = 52.42 ms, *SD* = 84.66).

No three-way interactions were found (*F* < 2.5).

### Comparing SC to controls on the priming task

The priming task was designed based on SC's report of her synesthetic experiences and therefore, we expected that she would exhibit behavior that was statistically different from that of a trained group of controls. We compared SC to the control group in order to objectively assess the statistical significance of her behavior in each condition. In order to compare SC's data to the control group and furthermore, restrict the number of comparisons made, we calculated the scores in accuracy and RT for each of the main effects and 2 × 2 interaction effects and tested whether SC's score was significantly different from that of the control group in each condition. One-tailed *t*-tests were used because we expected only larger effects for SC compared to controls for all conditions (i.e., we did not expect that effects would be larger in the control group). Significance levels were corrected for multiple comparisons using the Bonferroni method.

The results of the *t*-tests are reported in Table 3. No differences between SC and the control group were found for RT or accuracy. Therefore, we could not confirm our hypothesis that SC behaves significantly different on this task compared to a group of matched controls who had been briefly trained on a subset of SC's associations. For interpretation, the data of both SC and the control group is illustrated in Figure 3 (main effects) and Figure 4 (interaction effects).

**Table 3:** **Differences in performance on the priming between SC and controls for main effects and 2 × 2 interactions**.

**Dependent variable**	**Control sample**		**SC's score**	**Significance**		**Estimated % of the**		**Estimated effect**	
	**(***N* = 16**)**			**test**		**control population**		**size (*Z_cc_*)**	
						**obtaining a lower score**			
						**than the case**			
	**Mean**	***SD***		***t***	***p***	**Point**	**(95% *CI*)**	**Point**	**(95% *CI*)**
**EFFECT IN ACCURACY (%)**									
Visual vs. auditory	5.60	1.74	4.17	−0.80	0.219	21.89	(8.35 to 40.45)	−0.82	(−1.38 to −0.24)
Incongruent vs. congruent	−0.26	1.76	2.09	1.30	0.107	89.26	(73.97 to 97.75)	1.34	(0.64 to 2.01)
C−I vs. I−C direction	−8.99	1.94	−7.29	0.85	0.204	79.57	(61.24 to 92.59)	0.88	(0.29 to 1.45)
Modality × congruency	−0.26	2.07	4.17	2.08	0.028	97.23	(89.01 to 99.88)	2.14	(1.23 to 3.03)
Congruency × direction	2.08	3.99	2.08	0	0.500	50.00	(31.21 to 68.79)	0	(−0.49 to 0.49)
Modality × direction	11.20	2.78	10.42	−0.27	0.395	39.46	(21.88 to 58.86)	−0.28	(−0.78 to 0.22)
**EFFECT IN REACTION TIME (ms)**									
Visual vs. auditory	−42.23	94.76	−21.13	0.22	0.416	58.41	(39.08 to 76.28)	0.22	(−0.28 to 0.72)
Incongruent vs. congruent	17.09	58.37	−35.57	−0.88	0.198	19.76	(6.99 to 37.96)	−0.90	(−1.48 to −0.31)
C−I vs. I−C direction	155.84	88.19	4.79	−1.66	0.059	5.87	(0.65 to 17.84)	−1.71	(−2.48 to −0.92)
Modality × congruency	−65.71	79.72	−22.63	0.52	0.304	69.61	(50.26 to 85.52)	0.54	(0.01 to 1.06)
Congruency × direction	−10.48	73.64	−34.35	−0.31	0.379	37.87	(20.54 to 57.31)	−0.32	(−0.82 to 0.18)
Modality × direction	203.24	137.03	128.46	−0.53	0.302	30.21	(14.35 to 49.56)	−0.55	(−1.07 to −0.01)

Modality refers to the difference in the visual and auditory modalities. Congruency refers to the difference between incongruent and congruent prime-target taste relationships. Direction refers to the difference between the concurrent-inducer direction (C-I) and the inducer-concurrent direction (I-C) of the prime-target relationship. Tests were corrected for multiple comparisons. No significant differences (1-tailed) were found.

### fMRI activation

Whole brain *Z*-statistic values, MNI coordinates, cluster size, and brain regions are reported for each contrast of interest. Brain regions are based on the Harvard-Oxford Cortical Structural Atlas, the Juelich Histological Atlas, and Brodmann areas are reported from the Talairach Daemon when available.

### Written language localizer

In a passive-viewing localizer paradigm, SC was presented with four types of visual stimuli: written Dutch words, written Chinese characters, Dutch words that induced strong synesthetic experiences for SC (i.e., “tasty”), and Dutch words that induced weak-to-no synesthetic experiences for SC (i.e., “tasteless”).

**Figure 5:**
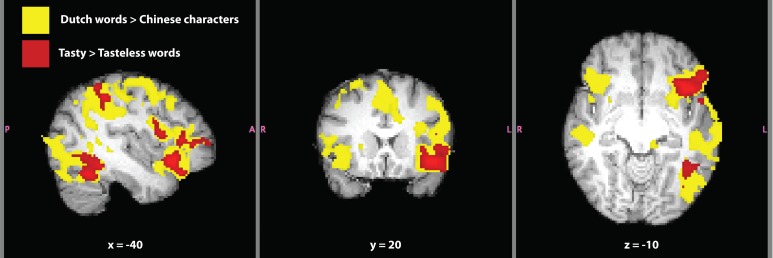
**Written language localizer for synesthete SC.** Significant activation for the contrasts *Dutch words* > *Chinese characters* (yellow) and *tasty* > *tasteless words* (red) are shown. Masks of whole-brain *Z*-statistic values are shown in MNI space, with SC's normalized brain as the background image. The mask in this image of the contrast *Dutch words* > *Chinese characters* represents the original level of cluster-based thresholding (*Z* > 2.3) not the *post-hoc* increased threshold of (*Z* > 3.1) and a corrected cluster-based significance threshold of *P* = 0.05.

The contrast *Dutch words* > *Chinese characters* is typically used to localize the VWFA in inferior temporal cortex, a brain region selective for the written form of words (e.g., Baker et al., 2007). Contrasting written language to unrecognizable written symbols is useful in order to isolate activation related to comprehension and semantics. The contrast revealed activation over much of SC's brain (Figure 5). Considering the range of functions involved in language comprehension and recognition, it is not surprising to find such widespread activation using our *a priori* threshold. Therefore, we increased the thresholding (cluster-based threshold of *Z* > 3.1 and corrected cluster-based threshold of *P* = 0.05) of this contrast *post hoc*, because of the large amount of activation found at the original level of thresholding. Significant activation and local maxima at the *post-hoc* threshold for the contrast of are given in Table 4. The VWFA was the second most significant cluster, after Broca's area, both in the left hemisphere. Activation in the first cluster encapsulated most of the left orbitofrontal cortex, the left supramarginal gyrus and extended into the left middle temporal gyrus. Activation unique to the left hemisphere was in extrastriate cortex and inferior occipital-temporal cortex (including the VWFA).

**Table 4:** **Significant clusters of fMRI activation in SC related to written language for the contrasts (A) *Dutch words* > *Chinese characters,* (B) *tasty* > *tasteless words*, and (C) *tasteless* > *tasty words***.

**Cluster**	**Voxels**	***Z*-max**	***X***	***Y***	***Z***	**Brain region**
DUTCH WORDS > CHINESE CHARACTERS^*^						
1	10,246	8.97	−46	16	16	L Inferior frontal gyrus BA 44/45 (“Broca's area”)
2	1392	9.04	−46	−62	−12	L Inferior temporal gyrus (“VWFA”)
3	1249	7.5	42	46	16	R Frontal pole BA 10
4	1188	5.94	50	−32	40	R Supramarginal gyrus
5	1102	10.2	−4	14	56	L Superior frontal gyrus BA 6
6	469	7.78	48	−28	−4	R Middle temporal gyrus
7	246	6.94	8	−76	−34	R Cerebellum
8	218	5.09	46	8	42	R Middle frontal gyrus
**TASTY > TASTELESS WORDS**						
1	2357	5.37	−56	12	2	L inferior frontal gyrus BA 44/45 (“Broca's area”)
1		4.99	−40	20	−10	L Frontal orbital cortex/insula
2	598	4.42	−42	−58	−22	L Temporal occipital fusiform/inferior temporal gyrus (“VWFA”)
3	415	4.14	−48	−42	54	L Parietal lobe/supramarginal gyrus BA 40
**TASTELESS > TASTY WORDS**						
1	2335	4.66	10	−52	34	R Precuneus/cingulate gyrus
1		3.79	4	−46	22	R Posterior cingulate gyrus
2	637	4.75	−32	−80	−38	L Cerebellum
3	508	4.62	26	50	36	R Frontal pole

* This contrast was thresholded (voxel-wise) post hoc using Z > 3.1 and a corrected cluster significance threshold of P = 0.05.

Whole brain Z-statistic values, MNI coordinates, cluster size, and brain regions are reported for each contrast of interest. Brain regions are based on the Harvard-Oxford Cortical Structural Atlas, the Juelich Histological Atlas, and Brodmann areas are reported from the Talairach Daemon when available. A higher cluster-based Z-threshold (Z > 3.1) was chosen post hoc for the contrast Dutch words > Chinese characters, because of the large extent of activation found at the original threshold (Z > 2.3). The corrected cluster significance threshold remained P = 0.05.

We next tested the comparison between written Dutch words that elicited a strong synesthetic experience (“tasty”) to written Dutch words that elicited weak-to-no synesthetic experience (“tasteless”; Table 3). This contrast is better suited to investigate brain activation in SC that was related to the synesthetic experience, because it subtracted out (most) differences in language that were present in the VWFA localizer. Significant activation for the contrast *tasty* > *tasteless* *words* (all written Dutch words) is presented in Table 4. This contrast yielded three distinct clusters in left hemisphere of SC's brain (Figure 5): a frontal region including inferior frontal gyrus, frontal pole, and orbitofrontal cortex along the anterior border with the insular cortex, an inferior temporal cluster corresponding to the VWFA, and a cluster in superior parietal lobe along the supramarginal gyrus. It should be noted that all three clusters in this contrast are overlapping entirely with areas in the contrast of *Dutch words* > *Chinese characters*, indicating that they are indeed part of SC's language processing network and not independent brain regions. The opposite contrast, *tasteless > tasty words*, again showed three significant clusters of activation that are presented in Table 4. These brain areas included right precuneal cortex next to the cingulate gyrus, right posterior cingulate cortex, left cerebellum, and right frontal pole (and notably did not overlap with activation in the other contrasts tested).

SC experiences smell in addition to taste. The whole-brain contrast *tasty > tasteless* words was inspected for evidence of activation in the primary olfactory cortex that is considered to be located in the piriform cortex (Gottfried et al., 2002; Small et al., 2005). We did not find significant activation that corresponded to the coordinates given for the frontal or temporal piriform cortices (Gottfried et al., 2002). Upon investigation of the uncorrected data, small clusters of activation in the left hippocampus and amygdala were found. The processing of odors involves several brain networks and furthermore, can be modulated based on context (Gottfried et al., 2002; Small et al., 2005). This experiment was not sensitive enough to separate olfactory and gustatory processes.

### Verbal and non-verbal sound localizer

In a passive-listening paradigm, SC was presented with four types of auditory stimuli: spoken Dutch, spoken foreign language, musical instruments, and environmental sounds. Sounds were localized in separate runs from visual stimuli, as SC was instructed to close her eyes during the presentation of auditory stimuli in the scanner. (Therefore, we cannot make a direct comparison between visually presented and spoken words).

**Figure 6:**
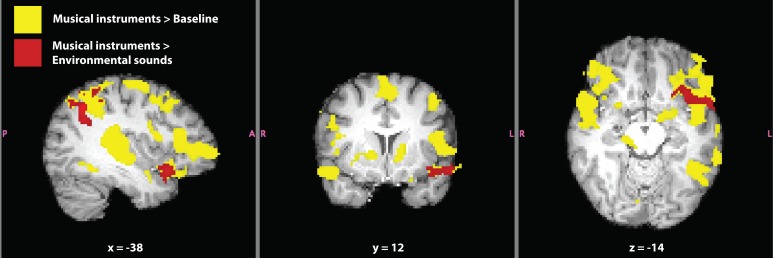
**Verbal and non-verbal language localizer for synesthete SC.** Significant activation for the contrasts *musical instruments* > *baseline* (yellow) and *musical instruments* > *environmental sounds* (red) are shown. The contrast *musical instruments* > *environmental sounds* represents a difference in synesthetic gustatory experience for SC (i.e., *“more”* > *“less” synesthesia*) in addition to differences in types of sounds. Masks of whole-brain *Z*-statistic values are shown in MNI space, with SC's normalized brain as the background image.

Significant activation and local maxima for the contrast of *native language* > *foreign language* are presented in Table 5. The contrast revealed activation over much of SC's brain (Figure 6) and in many of the same regions as the written language localizer. The analysis classified ten clusters of activation in both the left hemisphere and right hemispheres. Activation was seen bilaterally spreading across most of the middle temporal gyri from posterior to anterior regions, in inferior frontal gyrus (corresponding to Broca's area), in the superior and inferior parietal lobe along the supramarginal gyrus to the angular gyrus, and in orbitofrontal cortex (anterior to the insular cortex). The spoken language contrast also evoked activation in the left inferior temporal gyrus (SC's VWFA).

**Table 5:** **Significant clusters of fMRI activation in SC related to verbal and non-verbal sounds for the contrasts (A) native language > foreign language, (B) musical instruments > baseline, (C) environmental sounds > baseline, (D) musical instruments > environmental sounds, and (E) environmental sounds > musical instruments**.

**Cluster**	**Voxels**	***Z*-max**	***X***	***Y***	***Z***	**Brain region**
**NATIVE LANGUAGE > FOREIGN LANGUAGE (SPOKEN)**						
1	9649	10.5	−52	−36	−6	L Middle temporal gyrus
2	3465	9	50	−22	−12	R Middle temporal gyrus
3	1754	7.29	68	−32	18	R Superior temporal gyrus/supramarginal gyrus BA 42
4	1522	6.86	−52	18	24	L Inferior frontal gyrus BA 44/45 (“Broca's area”)
5	949	5.58	48	34	18	R Inferior frontal gyrus BA 45 (“Broca's area”)
6	868	4.61	−2	48	30	L Superior frontal gyrus BA 9
7	853	5.88	−44	24	−12	L Frontal orbital cortex
8	636	4.5	34	14	28	R Middle frontal gyrus/white matter
9	493	5.05	48	30	−12	R Frontal orbital cortex
10	467	5.26	−36	−48	−28	L Inferior temporal cortex/cerebellum
**MUSICAL INSTRUMENTS > BASELINE**						
1	17,623	15.3	−66	−30	14	L Superior temporal gyrus
2	5517	17	60	−26	8	R Superior temporal gyrus
3	3342	10.2	52	6	32	R Inferior frontal gyrus/pre−central gyrus BA 6
4	717	4.52	−12	10	−2	L Caudate nucleus
5	435	4.88	8	−78	−34	R Cerebellum
**ENVIRONMENTAL SOUNDS > BASELINE**						
1	5039	15.4	−56	−20	2	L Superior temporal gyrus
2	4871	15.3	60	−24	8	R Superior temporal gyrus
3	735	6	52	6	32	R Inferior frontal gyrus/pre−central gyrus BA 6
**MUSICAL INSTRUMENTS > ENVIRONMENTAL SOUNDS**						
1	924	4.44	−36	−60	42	L Intra−parietal sulcus
2	418	4	−18	30	50	L Superior frontal gyrus
3	407	4.17	−52	2	−8	L Superior temporal gyrus/anterior insula/orbital frontal cortex
4	383	5.58	−52	−42	20	L Angular gyrus
**ENVIRONMENTAL SOUNDS > MUSICAL INSTRUMENTS**						
1	758	5.52	54	−48	16	R Angular gyrus/inferior parietal lobe

Whole brain Z-statistic values, MNI coordinates, cluster size, and brain regions are reported for each contrast of interest. Brain regions are based on the Harvard-Oxford Cortical Structural Atlas, the Juelich Histological Atlas, and Brodmann areas are reported from the Talairach Daemon when available.

### Activation related to music and environmental sounds

The contrast *musical instruments* > *baseline* evoked activation in bilateral primary auditory cortex, orbitofrontal cortex (along the anterior border with insular cortex), inferior frontal gyrus, extending into Broca's area, and paracingulate gyrus (Figure 6). Additional activation was seen in the left hemisphere, in the superior parietal lobe along the supramarginal gyrus, and inferior temporal gyrus, overlapping with SC's VWFA (Table 5).

The contrast *environmental sounds* > *baseline* evoked activation in bilateral primary auditory cortex, and right inferior frontal gyrus near Broca's area. Activation related to environmental sounds was less widespread than that related to music, but it was almost entirely overlapping with the *musical instruments* > *baseline* activation (Table 5).

In the current paradigm, we were able to contrast the sounds of musical instruments to environmental noises. These contrasts related more directly to the difference in eliciting the synesthetic experience than that of spoken Dutch vs. spoken foreign languages. All of the musical instruments evoked synesthetic experiences in SC while only a few of the environmental noises did. In both conditions, SC rated that the sounds elicited a synesthetic experience of 3/5 (5 being the highest) for level of intensity. Therefore, this differed from the visual presentation of inducing vs. non-inducing synesthetic words related to the amount of stimuli that induced the synesthesia between contrasts. It should be noted that activation in this contrast is also related to differences between the musical and environmental stimuli, as they were not balanced for low-level properties such as tone and temporal frequency. Still, we believe that these stimuli are better matched to subtract out largely confounding effects, as those present in the spoken language contrast. We refer to the literature to identify consistent activations (in non-synesthetes) for these types of auditory stimuli.

Significant activation for the contrast *musical instruments* > *environmental sounds* is reported in Table 5 and shown in Figure 6. Four clusters of activation were found only in the left hemisphere: the superior parietal lobe around the supramarginal gyrus, angular gyrus, the superior temporal pole extending into the anterior insula and orbitofrontal cortex, and paracingulate gyrus in the frontal lobe. The opposite contrast, *environmental sounds* > *musical instruments* yielded activation in one cluster in the right angular gyrus (Table 5). The contrasts *musical instruments* > *environmental sounds* and *environmental sounds* > *musical instruments* yielded significant activation in left and right angular gyri, respectively. This cluster found in the angular gyrus of the right hemisphere was located posterior to the location of the cluster in the corresponding angular gyrus of the left hemisphere.

## Discussion

We present a case report on synesthete SC, who experiences lexical-gustatory and sound-gustatory synesthesia. In addition to gustatory sensations (~60% of words tested), ~30% of words induced smells, and ~10% of words induced another type of sensation or a combination of experiences. SC's synesthesia was stable over time and more consistent than a group of controls. Her long-term consistency for a large list of synesthetic inducers serves as the synesthetic “test of genuineness” and is comparable to previous reports of lexical-gustatory synesthesia (Baron-Cohen et al., 1987; Ward and Simner, 2003; Ward et al., 2005; Gendle, 2007; Simner and Haywood, 2009; Richer et al., 2011). SC exhibited behavior and reported experiences that are in line with previous reports of lexical-gustatory synesthesia. SC reported experiencing synesthesia her whole life; that it is induced by hearing people speak, speaking herself, reading and “inner” speech; the concurrent sensations are as complex as real tastes or smells, which can be pleasant and unpleasant; the concurrent sensations are felt on the tongue, mouth and throat; food words taste like the foods that they describe (Pierce, 1907; Ward and Simner, 2003; Gendle, 2007; Richer et al., 2011). SC shares some specific qualities with synesthete TD, who also reported going out of her way to avoid speaking “ugly words” that induce a very unpleasant taste concurrent. SC has concurrent sensations not entirely related to taste or smell, such as feelings of pressure and objects in the mouth, similar to Pierce's case study and synesthete PS. Still, differences between SC and what has so far been reported in detail in the literature exist. For instance, SC did not report experiencing temperature along with the concurrent sensations and experiences complex synesthesia for non-linguistic sounds, including musical instruments and environmental noises. Furthermore, SC experiences synesthesia for most of the words tested (94.5%), and this percentage varies widely between lexical-gustatory synesthetes: 46–100% across 5 synesthetes (Simner and Haywood, 2009), 31–86% across 7 synesthetes (Ward et al., 2005), 47.3% in synesthete TD (Gendle, 2007), 44% in synesthete JIW (Ward and Simner, 2003), 42% in synesthete PS (Richer et al., 2011), illustrating the range of inter-individual differences within a sub-type of synesthesia.

### Priming task

Based on the experiences reported by SC, a behavioral priming task was designed in order to test certain assumptions and to see if her behavior on this task stood out from a group of controls trained on a subset of SC's associations. In this priming task, participants indicated the type of taste of the target word (sweet, bitter, sour, or salty). The priming task was presented in both a visual and auditory modality. Furthermore, it was divided into two taste conditions and two word-pair directions, which refer to the relationship of the prime to the target word. In a congruent trial, the prime and target word had the same type of taste (e.g., sweet followed by sweet), while in an incongruent trial, the prime and target word had different types of taste (e.g., sweet followed by sour). The direction of the synesthetic inducer and concurrent was also balanced between the primes and target words. In an I-C direction trial, an inducer preceded a concurrent (e.g., *alsof* followed by *softijs*), while in a C-I direction trial, a concurrent preceded an inducer (e.g., *softijs* followed by *alsof*). We compared SC to the trained control group in order to objectively assess the statistical significance of her behavior in each condition.

Significant main effects of presentation modality (Figure 3A) and inducer-concurrent direction were found in the control group (Figures 3E,F). The main effect of congruency was not significant as expected, nor did it differ between SC and the controls (Figures 3C,D). However, congruency and presentation modalities interacted in such a way that the expected congruency effect (in RT) was present in the auditory modality but not in the visual modality for the control group (Figure 4B). SC did not differ significantly from controls in RT, however, SC performed better on incongruent trials in the visual modality (100%) compared to congruent trials (93.75%), and showed no difference between incongruent (93.75%) and congruent trials (93.75%) in the auditory modality. No such difference was present in accuracy for the control group. We expected SC to have higher accuracy on congruent compared to incongruent trials. The difference between SC and controls was not significant, nor did it differ as we expected. Therefore, any explanation for SC's higher accuracy on incongruent trials would be speculative at best. We conclude that the interaction between modality and congruency showed the closest dissociation in performance of SC compared to the group of controls.

An interesting and unexpected effect of inducer-concurrent direction in the control group was found (Figures 3E,F). The control participants were trained to associate the word pairs in both directions. Therefore, we did not expect an effect of inducer-concurrent direction to be present in the control group, and we expected that SC would differ significantly in this respect from the controls. In contrast to our expectations, SC performed similarly to the controls in both the I-C and C-I directions (Figure 3F). Accuracy was significantly higher in the I-C direction compared to the C-I direction (Figure 3E). This may be due to the fact that the original list from which the controls studied before training, as well as the paper test of the associations given after training, were only presented in the I-C direction. This may have been enough of an influence to cause the effect of direction in the controls. We note that one of the control participants spontaneously reported that the learned associations “feel natural” and noticed that the word pairs shared phonological properties, such as similar vowel tones. Associations between tastes and pitch are known to exist in non-synesthetes (Crisinel and Spence, 2009; Simner et al., 2010). Naturally occurring (but mainly unconscious) associations between the phonology of the word used and the foods that they were associated with may explain at least part of the effects found in the control group. However, this hypothesis is an interesting avenue for future research.

SC's performance on this priming task did not differ significantly from the control group on any main effects or interaction effects. We conclude that SC's behavior followed the same pattern as that of the control group who were trained to associate this subset of SC's word-taste pairs. When such a control group is used, then it is apparent that this task should not be considered as a diagnostic tool in assessing behavioral differences related to the presence of lexical-gustatory synesthesia. The similarities between SC and the trained control group on the priming task are quite striking. The controls were trained briefly, especially in comparison to SC's life-long experiences. They showed a very strong pattern of behavior on the priming task, where almost each effect reached significance in RT, accuracy or both. We did not, however, compare SC's performance to a group of untrained matched controls. SC's behavior did reflect that of the trained control group, which is objective evidence that she does have word-taste associations, as she was never trained to associate the word pairs to each other as the controls were. The word pairs used were SC's own associations, which she describes as automatic, consistent and having been present as long as she can remember. The question remains open why the behavior of a synesthete so closely resembles that of controls briefly trained on semantic associations. Perhaps the task design was insensitive to key elements of SC's synesthetic experience in contrast to the semantic nature of her associations.

### SC's brain activation

Using fMRI, we measured SC's brain activation while passively viewing and hearing different sets of stimuli. SC served as her own control in the fMRI experiment, as practical limitations prevented us from being able to scan a comparable control group. Therefore, the data presented here should be considered exploratory in nature. In order to better understand the brain activation related to SC's synesthesia, we employed blocked designs as well as control conditions to evaluate SC's brain activation related to well-known functions, such as language processing (Danckert and Mirsattarri, 2012). We conclude that SC's brain activation related to language (both written and spoken) was localized successfully compared to the known literature on language related activation (Fedorenko et al., 2010, 2011, 2012), evoking activity in well-known language related structures, such as bilateral middle temporal gyri, inferior frontal gyri, orbitofrontal gyri, including Broca's area, and inferior temporal cortex in the fusiform gyrus (Figure 5). In addition, we tested whether SC showed normal brain activation upon hearing musical instruments and environmental sounds compared to baseline (no stimulation). We can conclude that SC showed normal brain activation upon hearing non-linguistic auditory stimuli (Fedorenko et al., 2011). Activation related to musical instruments was more extended compared with environmental sounds. Activation related to environmental sounds was contained within regions activated by musical instruments (both whole-brain contrasts), implying a shared neural substrate between these categories of sound. These shared regions were bilateral superior temporal gyri and right inferior frontal gyrus (the region of the right hemisphere that corresponds to Broca's area in the left hemisphere).

A comparison between the “tasty” to “tasteless” stimuli allowed us to localize brain activation related to SC's synesthetic experiences by canceling out most of the activation related to language comprehension, because some visually presented words hardly induced any synesthesia in SC. Three clusters of activation were found in the whole-brain analysis (all in the left hemisphere): inferior frontal cortex (Broca's area) extending into orbitofrontal and anterior insular cortex, the VWFA in inferior temporal cortex, and the parietal lobe along the supramarginal gyrus (Figure 5). Interestingly, all these clusters of activation are “contained” within the activation found for written language, implying that the neural substrate of SC's synesthetic experience is not independent from the neural substrate of language. As we were not able to test a control group, we recognize that activation in these contrasts may not be entirely related to synesthesia. We present SC's activation as a supplement to Jones et al. (2011) so that it may serve as a useful reference for future fMRI studies on such rare cases of lexical-gustatory synesthesia.

The contrast used here (*tasty* > *tasteless words*) is similar but not entirely comparable to the one reported in the categorical analysis by Jones et al. (2011) for participant JIW. Jones et al. compared pleasant, unpleasant, and emotionally neutral words (balanced for frequency, age of acquisition and number of syllables) that induced tastes to tasteless words. In the current study, SC rated words as either pleasant or unpleasant as a *post-hoc* analysis; therefore, emotional affect was not balanced in the design. Due to the fact that SC experiences synesthesia for almost every word she hears, stimuli were not balanced for frequency, age of acquisition or number of syllables.

Significant whole-brain activation for JIW was found in the right middle occipital gyrus and left lingual gyrus for the contrast *tasty* > *tasteless* *words* (Jones et al., 2011). We did not find corresponding activation for SC for the *tasty* > *tasteless* *words* contrast. When JIW's brain activation for the *tasty* > *tasteless* *words* contrast was compared to a control group, a difference in activation was found in JIW's precuneal cortex along the central commissure. We found precuneal cortex activation in SC's brain, but for the opposite contrast (whole brain) of *tasteless* > *tasty* *words*. The precuneal cortex is implicated in a wide variety of cognitive functions, such as integration of multimodal information and has shown up in both functional and structural imaging studies on grapheme-color synesthesia (Jones et al., 2011). The increase in activation in response to tasteless words compared to tasty words in these regions may reflect an interaction between processes that reflect the (missing) internal experience of the synesthetic concurrent and not the content of that experience. Alternatively, such activation could be reflective of default mode network activation (Fransson and Marrelec, 2008). Further research is needed as these findings were not expected and the explanations are purely exploratory.

Jones et al. (2011) used three regions of interest (ROIs) defined *a priori* based on their role in processing taste and affective components of taste (pleasant vs. unpleasant vs. neutral). These were anterior insula, orbitofrontal cortex, and anterior cingulate. In our whole brain analysis of SC, we found activation in the left orbitofrontal cortex along the border with the anterior insula. Jones and colleagues found that activation in this region related to the emotional content of the taste compared to neutral tastes, and specifically to unpleasant tastes. In JIW's data, pleasant and unpleasant words elicited more activity in left anterior insular cortex than words that induced affectively neutral tastes. The contrast of *unpleasant > neutral* stimuli differed between JIW and the control group in the anterior insula. We did not separate or balance conditions of stimuli based on SC's affective evaluation of the concurrent sensations, but *post-hoc* inspection validated the fact that both pleasant an unpleasant synesthetic experiences were related to the words used as stimuli (64% were pleasant).

Unlike JIW, SC experiences synesthesia induced by non-linguistic and non-vocal sounds (Ward and Simner, 2003). Therefore, we were presented with a unique opportunity to investigate brain activation related to sound-gustatory synesthesia in SC. All the musical instruments used as stimuli induced a concurrent sensation in SC, while only a few of the environmental sounds did. Although these types of stimuli were not balanced to account for low-level differences in acoustic properties, we contrasted musical instruments to environmental sounds in order to better localize activation related to differences in SC's synesthetic experience (Figure 6). Furthermore, the fact that these types of sounds share a neural substrate implies that the same brain regions share functional mechanisms. The contrast *musical instruments* > *environmental sounds* can be compared to the contrast *tasty* > *tasteless* *words* (written form), because both contrasts involve a difference between “more” and “less” synesthetic experiences for SC (although these contrasts are not equivalent). We visually compared the contrast *musical instruments* > *environmental sounds* to the contrast *tasteless* > *tasty* *words* for written words, and we found that two regions of activation were overlapping: the left superior parietal cortex along the supramarginal gyrus and left anterior insular cortex along the border of the inferior orbitofrontal cortex. The primary gustatory cortex is located in the anterior insular cortex (Ogawa et al., 2005; Veldhuizen et al., 2007; Small, 2010), and this activation may reflect SC's synesthetic experience of taste or the affective component of this experience. The region in the superior parietal lobe is activated at the group level and across studies of grapheme-color synesthesia, and a common role in the conscious and attentional binding of components of sensory stimuli across sub-types of synesthesia has been proposed (Rouw et al., 2011).

We cannot conclude that we have localized activation related to synesthesia alone, as we had no control group to compare SC's activation to. However, based on what is known about gustatory, linguistic, auditory and synesthetic brain activation, in addition the only other (known) publication on brain activation in a lexical-gustatory synesthete (Jones et al., 2011), we feel that the data obtained on SC was reliable and reflective of her synesthesia. Taken together these results showed that the synesthetic experience of taste was not only reflected in the brain activation of a gustatory-type synesthete, but furthermore, that the modality of the inducer (visual-lexical, auditory-lexical, and non-lexical auditory stimuli) could be differentiated from these patterns of brain activity.

## Conclusion

We were presented with a rare opportunity to investigate a case of lexical-gustatory and sound-gustatory synesthesia in SC. The authenticity of her experiences are reflected in the consistency of her word-taste associations as well as during an objective behavioral priming task, although, not always in ways that were expected. Such a rare form of synesthesia provides us with the opportunity to understand neural differences at the individual level that underlie differences in phenomenological experiences.

### Conflict of interest statement

The authors declare that the research was conducted in the absence of any commercial or financial relationships that could be construed as a potential conflict of interest.
